# Accurately fitting biophysical neuron models to experimental voltage data enabled by meta-learning

**DOI:** 10.21203/rs.3.rs-9694895/v1

**Published:** 2026-06-02

**Authors:** Roy Ben-Shalom, Kyung Geun Kim, Alexander Ladd, Prarthan Ghosh, Timothy Fenton, Henry Kyoung, Ghazaleh Yazdani, Kaustubh Chakravarthula, Gunther H. Weber, Caleb Geniesse, Tiankai Xie, Kevin J. Bender, Kristofer E. Bouchard

**Affiliations:** 1 MIND Institute, University of California, Davis, Sacramento, CA, USA; 2 Department of Neurology, University of California, Davis, Sacramento, CA, USA; 3 Department of Bioengineering, Stanford University, Stanford, CA, USA; 4 Department of Electrical Engineering and Computer Sciences, University of California, Berkeley, Berkeley, CA, USA; 5 Department of Neurobiology and Biophysics, University of Washington, Seattle, WA, USA; 6 Scientific Data Division, Lawrence Berkeley National Laboratory, Berkeley, CA, USA; 7 Department of Neurobiology, Harvard Medical School, Boston, MA, USA; 8 School of Computing and Augmented Intelligence, Arizona State University, Tempe, AZ, USA; 9 Weill Institute for Neurosciences, University of California, San Francisco, San Francisco, CA, USA; 10 Department of Neurology, University of California, San Francisco, San Francisco, CA, USA; 11 Redwood Center for Theoretical Neuroscience, University of California, Berkeley, Berkeley, CA, USA; 12 Helen Wills Neuroscience Institute, University of California, Berkeley, Berkeley, CA, USA; 13 These authors contributed equally: Roy Ben-Shalom, Kyung Geun Kim, Alexander Ladd

## Abstract

The neuron is a fundamental unit of computation in the brain. Neuronal firing properties are governed by ion channels distributed across complex morphologies. Determining ionic conductances from experimental observations of intracellular membrane potentials would advance basic understanding and clinical translation, but is currently considered intractable. To enable accurate fitting of biophysical neuron models to experimental somatic voltage recordings, we developed a meta-learning algorithm (‘CoMParE’). Using CoMParE to fit standard biophysically detailed models, we demonstrate state-of-the-art reproduction of experimental data and quantify parameter estimate precision and accuracy. We additionally fit models with enhanced electrophysiological detail and demonstrate further improvements in experimental data reproduction. Finally, we show that these improvements result from convexifying the objective function loss surface through meta-learning. These results demonstrate that highly detailed biophysical models can accurately reproduce experimental data, enabling determination of ionic conductances underlying neuronal function and neurological disorders, and indicating that this inverse problem is tractable.

## Introduction

**Figure 1.**
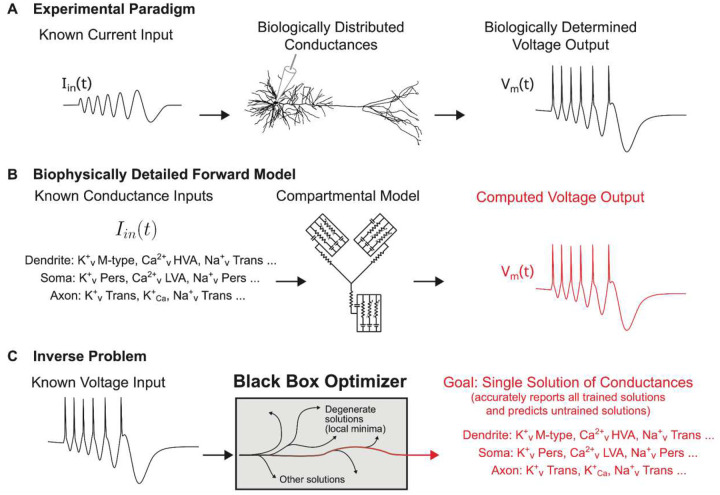
Determining ionic conductances from membrane potential recordings is an inverse problem. A: Single-cell electrophysiological experiments deliver a known time-varying current stimulus (left, *I*_*in*_(*t*)) to the soma of a neuron in current-clamp mode (middle) to record the elicited experimentally determined membrane potential (right, *V*_*m*_(*t*)). B: Biophysically detailed multicompartment neuron models specify the values of ionic conductances (e.g., Na^+^/K^+^/Ca^+^) and membrane properties (e.g., R_m_) distributed across multiple dendritic, somatic, and axonal compartments (left). Given a known current injection stimulus (left, *I*_*in*_(*t*)), the multi-compartment RC-circuit model (middle) with the specified conductances produces computationally determined membrane potentials (right). C: The inverse problem of taking an experimentally determined membrane potential (*V*_*m*_(*t*)) in response to current injection (*I*_*in*_(*t*)) and using a black box optimizer (middle) to fit the parameters (e.g., conductances, left) of a biophysically detailed multicompartment model is impeded by the existence of degenerate solutions

Understanding the inner workings of single neurons is fundamental to our understanding of the nervous system. The remarkable spectrum of computational functions performed by neurons, from coincidence detection to temporal integration, result from diverse neuronal morphologies and precisely orchestrated ionic conductances that together determine membrane voltage dynamics (V_m_(t)) from transmembrane currents (I_in_(t), Fig.1A) (Softky and Koch, 1993; Hausser, Spruston and Stuart, 2000). Importantly, disruption of ion channels (channelopathies) are a major contributor to many neurological disorders (Kullmann, 2010; Hedrich, Lauxmann and Lerche, 2019; Kessi *et al*., 2020, 2021). Unfortunately, careful empirical determination of the complement and distribution of conductances across a neuron’s morphology is experimentally laborious, and has been performed on relatively few neuronal classes (Major, Larkum and Schiller, 2013; Almog and Korngreen, 2014; Gidon *et al*., 2020). Indeed, it may be intractable to experimentally acquire the datasets necessary to fully understand the computational implications of the large diversity of cortical neurons at the microscopic scale of ionic conductances (Golowasch *et al*., 2002; Bano-Otalora *et al*., 2021). This gap impedes our fundamental understanding of the inner workings of neurons. Translationally, high-throughput determination of conductances would aid in understanding the etiology of diverse human illnesses that affect the integrative properties of neurons, including many forms of neurodevelopmental and neuropsychiatric disorders.

The development of computational models initially offered promise for addressing this challenge. The seminal work of (Hodgkin and Huxley, 1952) determined the biophysical basis of action potential generation and Wilfrid Rall’s cable theory (Rall, 1962; Rall *et al*., 1992) provided first-principles description of electrical propagation across neuronal compartments (Hille, 1984) (Fig.1B). The patch-clamp technique revolutionized this endeavor by enabling direct measurement of ionic currents and precise voltage recordings from individual neurons, providing the quantitative experimental constraints necessary to parameterize a neuron (Sakmann and Neher, 1984). Separately, efficient computational implementations of biophysically detailed neuron models such as NEURON (Hines, 1984, 1993) made it feasible to simulate complex multi-compartmental models incorporating realistic morphologies and heterogeneous ion channel distributions, transforming theoretical cable equations into practical tools for modeling biological neurons (Mainen *et al*., 1996). These works drove considerable optimism that biophysically detailed multi-compartmental models (Fig. 1B) could be used to determine ionic conductances from experimentally accessible membrane potential recordings.

Yet as these methods matured, they exposed a deep mathematical challenge. Attempting to infer underlying ionic conductances (microscopic variables) from the ensemble “readout” of intracellular V_m_(t) (macroscopic variable) is an example of an inverse problem. Current thought in neuroscience is that most inverse problems are mathematically degenerate/intractable; i.e., many configurations of conductances are thought to give rise to identical membrane potentials (Fig. 1C). Much of our thinking about inverse problems in neuroscience is shaped by the seminal work of (Prinz, Bucher and Marder, 2004), which demonstrated that broad families of single neuron parameter values produce similar pyloric rhythms in the crab stomatogastric ganglion circuit. Thus, the inverse problem of determining microscopic properties of neurons from features of spike-trains in networks of neurons seems to be degenerate: multiple configurations of an individual neuron’s properties can give rise to similar network rhythms (Prinz, Bucher and Marder, 2004; Marder and Goaillard, 2006).

Efforts to fit parameters in complex single neuronal models face similar difficulty, leading many to speculate that, even at the single neuron level, the vast majority of conductances are redundant with respect to producing V_m_ (Hay *et al*., 2011; Bahl *et al*., 2012; Almog and Korngreen, 2016; Gouwens *et al*., 2018). Evolutionary Algorithms (EA) are the current state-of-the-art approach for fitting parameters of biophysically detailed single-neuron models (Druckmann *et al*., 2007; Almog and Korngreen, 2014; Van Geit *et al*., 2016; Ladd *et al*., 2022). However, current approaches to EA for fitting single-neuron models suffer several limitations. Most detrimentally, the results (i.e., inferred channel parameters) depend heavily on the *ad hoc* objective function metrics (AP shape, interspike interval, afterhyperpolarization, etc.) used to compare the model’s output to the target V_m_(t) data, with different combinations of features/metrics often producing different results (Golowasch *et al*., 2002; Druckmann *et al*., 2013; Almog and Korngreen, 2016; Gouwens *et al*., 2018). These difficulties and considerations have further resulted in reduced effort to build more sophisticated and accurate neuron models.

In contrast to the predominant view, we hypothesized that the apparent degeneracy may not reflect biological reality but rather a failure of our computational approaches (Fig. 1C), and in particular the use of *ad hoc* objective functions. To test this hypothesis, we developed CoMParE (Conductance-based Model Parameter Evaluation), a novel meta-learning framework. Rather than relying on manually selected features with *ad hoc* weightings, CoMParE meta-learns weighted combinations of electrophysiological features that together form effective objective functions for fitting model parameters (e.g., conductances) across diverse current injection protocols. Using standard Blue Brain Project (BBP) neuron models (Markram *et al*., 2015; Ramaswamy *et al*., 2015), we demonstrate that CoMParE achieves superior reproduction of experimental data compared to state-of-the-art methods. Additionally, we quantify the precision and accuracy of inferring ionic conductances from somatic membrane potential measurement. We further developed a new NEURON (Awile et al., 2022) multi-compartmental model with additional ion channel subtypes and demonstrated that, when fit with CoMParE, this model yields better matches to experimental data. To provide insights into these improvements, we show that CoMParE reshapes the objective function loss surface to remove spurious critical points (e.g., minima) through meta-learning. Together, these results challenge the prevailing view that the vast majority of conductances are degenerate with respect to producing V_m_(t) in recorded compartments (e.g., soma). Our results demonstrate that specific values of multiple conductances combine to produce specific membrane potential dynamics. This advance has immediate implications for understanding neuronal diversity, computational specialization, and the etiology of channelopathies underlying neurological disorders.

## Results

The Conductance-Based Model Parameter Evaluation (CoMParE) method sculpts objective functions through meta-learning.

Our goal was to enable accurate fitting of biophysically detailed neuron models to commonly acquired experimental data of somatic membrane voltage (V_m_(t)) recordings in response to current injection I_in_(t) (below, we drop the time-dependence for notational simplicity). To this end, we developed CoMParE (Conductance-based Model Parameter Evaluation), a novel meta-learning framework that utilizes simulations to (meta-) learn objective functions that maximize the relationship between objective function value (i.e., error in model reproduction of observed V_m_) and ground truth values of model parameters. Such an objective function could make the problem of fitting those neuronal models to experimental data (with unknown parameters) more accurate and hence improve reproduction of V_m_.

The central innovation of CoMParE is to meta-learn objective functions (‘error functions’ that quantify the difference between a ‘target’ V_m_ and a ‘model’ V_m_) that improve fitting of neuron model parameters. Conceptually, CoMParE transforms the rugged landscapes of *ad hoc* objective functions that calculate model error relative to a target (Err) into smoother landscapes where optimization converges closer to ground-truth parameters. This is done by finding linear weighted combinations of electrophysiological features (eFEL+ features, for illustration see Fig. S1) that characterize multi-scale structure of V_m_ traces. Further analysis of eFEL+ feature covariance shows that eFEL+ features exhibit functional clustering (Fig. S2). CoMParE finds weights on eFEL+ features maximize the correlation (a positive-monotonic relationship) between error (Err) and model parameter distance from ground-truth parameter values (distance rank, ‘DR’) (quantified as Spearman Rho, ρ(Err, DR)).

For a given model (e.g., Hodgkin-Huxley) with target parameters, CoMParE creates many surrogate V_m_ traces with diverse model parameter set values in response to diverse somatic current injection protocols (I_in_). These surrogate V_m_s are compared to the target V_m_ (i.e., Err is calculated) as is the ranked distance of the surrogate model parameters to the target parameters (i.e., DR is calculated) (see Methods). With the sampling of model parameters, stimulus protocols, and eFEL+ features, CoMParE then finds an optimized linear combination of weights across all features to construct a single objective function that has the highest correlation between Err and DR (i.e., maximize ρ(Err, DR)) across all stimuli. This meta-learned objective function facilitates effective optimization of neuron model parameters (e.g., ionic channel conductances) from commonly collected observations of somatic V_m_. We note that CoMParE makes no guarantees of identifiability of all parameters of a model; indeed, depending on the model, some parameters may be inherently ill-defined with respect to somatic V_m_ for any objective function. The final step of CoMParE is using the meta-learned objective function for subsequent optimization (with EA) of the neuron model with respect to experimentally collected data V_m_. See Technical Box and Methods for further details.

**Figure 2:**
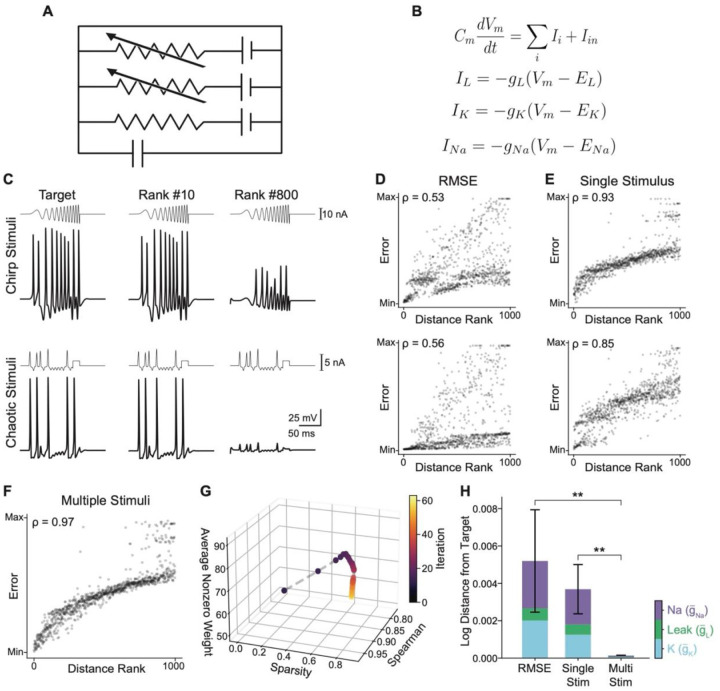
The Conductance-Based Model Parameter Evaluation (CoMParE) method sculpts objective functions through meta-learning. A-B: Circuit diagram and differential equation associated with the 3 parameter Hodgkin-Huxley (HH) model. C: Voltage responses of the HH model with target parameter set (global minimum) and sampled parameter sets at rank 10 (close) and rank 800 (far) with two different stimuli (chirp: top and chaotic: bottom). D: Error-rank plots for the chirp and chaotic stimuli with 1000 parameter set samples with HH model and root mean square error (RMSE) as an objective function. E: For both stimuli in C, linearly combining eFEL+ features with meta-learning (single-stim) results in higher Spearman rank-correlation coefficient than RMSE alone. F: Combining multiple eFEL+ features with meta-learning across multiple stimuli (multi-stim) results in nearly perfect Spearman correlation coefficient with *ρ* = 0.97. G: Trajectory during multi-stimulus meta-learing, plotting average nonzero weights vs. sparsity vs. Spearman coefficient. H: Log-normalized distance (mean and standard error across N = 10 random seeds of EA; **p < 0.01, Wilcoxon signed-rank test) from the ground truth (target) parameters of the HH model fitted with RMSE, CoMParE single-stimulus, and CoMParE multi-stimulus objective functions.

As a first demonstration of CoMParE’s ability to fit neuron models, we consider the single-compartment Hodgkin-Huxley (HH) model of the electrophysiological mechanism of action potential generation (see Methods). The equivalent RC circuit for this model is displayed in Figure 2A, while the corresponding generating equations are displayed in Figure 2B. Here, the three conductances (*g*_*k*_, *g*_*L*_, *g*_*Na*_) are the parameters to be fit to somatic V_m_ data for a given current injection. Figure 2C shows example V_m_ evoked by two stimuli (chirp, top; chaotic, bottom) for the ground truth model (left) as well as the rank 10 (center) and rank 800 models (right). The parameters associated with the rank 10 model clearly generates V_m_ that is a closer reproduction of the ground truth for both stimuli compared to the rank 800 model. Figure 2D shows error vs. rank distance plots when using a single error feature for these two stimuli (the RMSE between the ground truth and fit model for each stimulus), which resulted in only a modest correlation between error and distance rank (ρ = 0.53, 0.56). Figure 2E shows a CoMParE-meta-learned error function for the same single stimulus (single-stim), which improved the correlation substantially (ρ = 0.85, 0.93). Finally, Figure 2F shows that by meta-learning across eFEL+ features and stimuli (20 stims) achieves near-perfect monotonicity across multiple stimuli (multi-stim, ρ = 0.97).

The results above indicate that CoMParE meta-learning transformed the rugged objective function landscape into a smooth, more convex surface where parameter distance rank strongly predicts error. To understand the process of meta-learning the objective function, Figure 2G plots a meta-learning trajectory from CoMParE. Early iterations (dark colors) show relatively low Spearman correlation (ρ < 0.8), and as optimization progresses (lighter colors) correlation increases, reaching a final value of ρ = 0.97. Concomitantly the weight sparsity (i.e., the number of features with 0 weight) and the average of nonzero weights show qualitatively similar behavior, quickly increasing in the early iterations and then slowly converging in the later iterations. These behaviors suggest that the algorithm first explores broadly, then focuses on the most important features.

To demonstrate the impact of improved objective functions on model fit quality, Figure 2H displays the cumulative and per parameter error (log-normalized distance, see Methods) for the RMSE (e.g., Fig. 2D), meta-learned single-stim (e.g., Fig. 2E), and multi-stim (e.g., Fig. 2F) objective functions. The multi-stim meta-learned objective function achieved nearly perfect parameter fitting, and was significantly better than the other two (p < 0.01, Wilcoxon signed-rank test, WSRT, N = 10 random seeds of EA). Together, these results suggest that the CoMParE meta-learned objective function would improve the quality of optimized parameters of biophysical models fit to experimentally observed V_m_ data.

CoMParE enables accurate parameter estimation in a biophysically detailed neuron model from simulated data.

**Figure 3.**
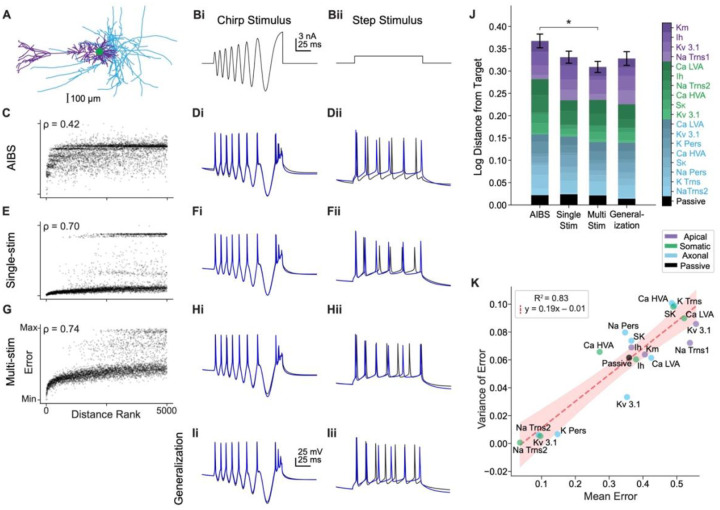
CoMParE enables accurate parameter fitting in a biophysically detailed neuron model from simulated data. A: Morphology of the BBP model. Bi: Chirp stimulus used to fit model parameters using EA. Bii: Step stimulus used to evaluate the fitted model. C: Error vs. distance rank of AIBS objective function. Di-Dii: Voltage responses (using stimulus Bi, Bii) from the fitted model using AIBS objective function. E: Error vs. distance rank plot single-stimulus CoMParE-meta-learned objective function. Fi-Fii: Voltage responses (using Bi, Bii) from the fitted model using CoMParE-meta-learned single-stimulus objective. G: Error vs. distance rank plot multi-stimulus CoMParE-meta-learned objective function. Hi-Hii: Voltage responses (using Bi, Bii) from the fitted model using CoMParE-meta-learned multi-stimulus objective. Ii-Iii: Voltage responses (using Bi, Bii) from the fitted model using the multi-stimulus CoMParE objective (same function as G) but the target responses during EA were generated with different model parameters of the BBP model to test generalizability. J: Log-normalized distance (mean and standard error across N = 20 random seeds of EA) from the ground-truth (target) of all parameters for every fitted model using AIBS, single-stimulus CoMParE, multi-stimulus CoMParE and generalizability test setting. K: Variance of error vs. mean of error plot for every model parameter (log-normalized distance from the target parameter) across N = 20 random seeds of EA.

The results above demonstrate that CoMParE enables accurate fitting of simple neuron models. However, more biophysically realistic models, such as the Blue Brain Project (BBP) models, express many more parameters and have far more complicated morphologies. This class of models is able to more robustly reproduce diverse V_m_ phenomena than simpler ones. Because the BBP model parameters are known (Ramaswamy *et al*., 2015), we can use them to generate surrogate waveforms to various stimuli for meta-learning of CoMParE and then use CoMParE to infer the model parameters. We used a model of a Layer 5 thick tufted pyramidal cell (L5TTPC) featuring realistic morphology and 24 parameters spanning multiple ion channel types and morphological segments (Fig. 3A, see Methods). Figure 3Bi–ii shows chirp and step stimuli of which Fig.3Bi shows a stimulus used in fitting while Fig.3Bii was not used in any optimization and thus was used to evaluate generalization.

We evaluated the ability of three different objective functions to fit the 24 parameters of this model from the V_m_ traces: (1) the *ad hoc* combination of 12 features used in Gouwens et al. (current state-of-the art, Allen Institute for Brain Science, AIBS) (Fig. 3C) (Gouwens *et al*., 2018), (2) CoMParE-meta-learned objective function for a single stimulus (Fig. 3E), and CoMParE-meta-learned objective function across multiple stimuli (Fig. 3G). Utilizing multiple stimuli increases the effective dimensionality of the objective function (Fig. S3), reducing parameter degeneracy by requiring fitted models to reproduce V_m_ across diverse conditions. The membrane potentials evoked by the stimuli in Figure 3Bi–ii for models fit with these objective functions are displayed in Figure 3D, F, H i–ii. We found that the quality of V_m_ generated by the models (grey traces) improved relative to the target (black traces), in particular for the multi-stimulus objective function evaluated on the held-out stimulus (panels ii). A critical test of CoMParE is whether the meta-learned objective function generalizes beyond the specific parameter sets used during meta-learning. Figure 3I shows V_m_ for the multi-stimulus CoMParE objective function applied to an unseen target voltage response generated with a different set of BBP model parameters.

We next examined the values of the fit parameters and found that CoMParE meta-learned objective functions enabled more accurate parameter fits. Figure 3J shows distance from target parameters (log_100_ of normalized distance) for each objective function (mean and standard error across N = 20 random seeds of EA, see Methods, see also Fig. S4 for learning curves). We found that the model optimized with *ad hoc* objective function (AIBS) had the lowest accuracy of learned model parameters. The single-stimulus CoMParE meta-learned objective shows some improvement, while the multi-stimulus CoMParE meta-learned objective demonstrates the highest accuracy (17% improvement from AIBS, ratio of log distances). Notably, the generalization condition (Fig. 3J) achieved comparable parameter accuracy to the multi-stimulus fit, confirming the result in Figure 3I. This generalization indicates that CoMParE meta-learned objective functions more robustly captures the model’s parameter-to-voltage mapping rather than features specific to a single parameter configuration. This generalizability is essential for applying CoMParE to experimental data where ground-truth parameters are unknown.

Out of widely available eFEL+ features, we found that small subsets were chosen repeatedly across diverse stimuli, but at the same time weight distribution varied across them (Fig. S5). Additionally, the meta-learning dynamics for this BBP model were qualitatively similar to that for the HH model, indicating that it is a general phenomenon (Fig. S6). Finally, to examine the robustness of individual parameter fits across different algorithm initializations (across N = 20 random seeds of EA, see Methods) we examined how variability in parameter error scales with the average error (Fig. 3K). We found a robust positive correlation between average per parameter error and the variability of that error (R^2^ = 0.83, N = 19 parameters, p < 0.001) with the conductances involved with action potential generation (e.g., axonal/somatic transient Na channels) being most robustly fit. Together, these results demonstrate that the CoMParE-meta-learned objective is able to accurately infer the ground truth parameters of the BBP model using only the model voltage responses.

**Figure 4.**
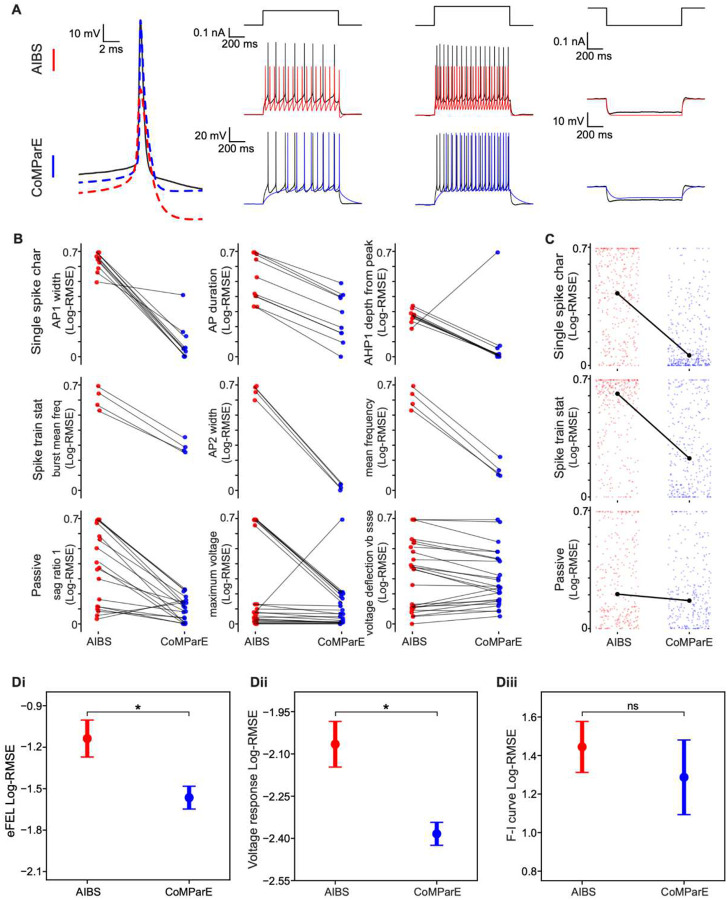
CoMParE fits biophysically detailed neuron models to accurately reproduce experimental data. A: Examples of single (median) spike, spike train, and passive responses resulting from three step stimuli for AIBS procedure and CoMParE using BBP model. B: Log root-mean-squared-error (log-RMSE) of selected electrophysiological features between a selected experimental cell response and model response for all stimuli. Each dot represents one stimulus, and gray lines connect the same stimulus across the two fits. C: Log-RMSE of all electrophysiological features across all stimuli for a selected cell. Each dot represents one stimulus–feature pair, and black dots connected by lines indicate the median log-RMSE across all pairs for the two fits. Di: Mean log-RMSE of all eFEL+ features across N = 10 fitted cells. Dii: Mean log-RMSE of raw voltage response traces across N = 10 fitted cells. Diii: Mean log-RMSE of frequency-current (F-I) curves across N = 10 fitted cells. Error bars indicate standard error (s.e.). ns, not significant; *p < 0.05, Wilcoxon signed-rank test.

The results above demonstrate that CoMParE improves accuracy in inferring biophysical parameters from data generated by neuron models with known ground truth parameters. To evaluate CoMParE’s performance on real experimental data, we fit the same BBP neuron model (see Methods) to N = 10 Layer 5 pyramidal cells from the Allen Cell Types Database, using both CoMParE and the AIBS standard fitting pipeline (Gouwens *et al*., 2018)Both approaches optimized the same set of biophysical parameters, allowing direct comparison of the resulting fits (Fig. 4).

Figure 4A displays typical experimental stimuli and the resulting voltage responses from a single experiment (black), as well as the AIBS (red) and CoMParE (blue) fit models. The median spikes are overlayed on the far left. Visually, models optimized with the CoMParE objective function produced V_m_ that more closely matched the ‘active’ response properties of the experimental data, in particular the action potential waveform (far left) and the temporal pattern of evoked spikes (middle two). The difference was more modest for the passive properties (far right) (note that the AIBS approach has a separate step for fitting just passive properties) (Gouwens *et al*., 2018). Figure 4B provides a quantitative evaluation across three different categories of electrophysiological features for these fits (each dot represents one stimulus, and gray lines connect the same stimulus across the two fits). The top row shows features describing single action potential (AP) characteristics (e.g., AP width, AP duration, and AHP depth from peak), the middle row examines spike train statistics (e.g., burst mean frequency, 2nd action potential width, and mean frequency), while the bottom row focuses on passive properties (e.g., sag ratio, maximum V_m_, and V_m_ deflection). Figure 4C presents quantification for all eFEL features in each feature category and for all stimuli (each dot represents one stimulus–feature pair, and black dots connected by lines indicate the median across all pairs for the two fits). These results demonstrate general improvement of CoMParE over the AIBS approach across diverse stimuli and V_m_ features (median log-RMSE CoMParE vs. AIBS, Single Spike: 0.06 vs. 0.43, Spike Train: 0.23 vs. 0.61, Passive: 0.17 vs. 0.20).

Similar results were observed across all N = 10 neurons we examined. Figure 4D shows aggregated performance across three different evaluation criteria for the N = 10 cells. Figure Di shows the mean error (log-RMSE) of every eFEL+ feature (all features contributing to the optimization process) across the cells (CoMParE vs. AIBS: −1.57 ± 0.08 vs. −1.14 ± 0.13, mean ± s.e., p < 0.05, N = 10, WSRT). Figure Dii shows the mean error (log-RMSE) between raw voltage traces of experimental and simulated responses (not an explicit part of the optimization process) (CoMParE vs. AIBS: −2.38 ± 0.04 vs. 2.07 ± 0.08, mean ± s.e., p < 0.05, N = 10, WSRT). Finally, Figure Diii shows error (log-RMSE) between firing-rate vs. current injection (F-I) curves of experimental and simulated responses (CoMParE vs. AIBS: 1.29 ± 0.19 vs. 1.44 ± 0.13, mean ± s.e., p > 0.05, N = 10, WSRT). These results demonstrate that CoMParE sets a new standard for state-of-the-art fitting of biophysically detailed models of neurons to reproduce experimental data.

In the previous section, we showed that CoMParE was able to fit neuron models that achieved substantial improvements in the accuracy of reproduced experimental data. We hypothesized that improving the objective function also enabled CoMParE to fit increasingly realistic, but therefore more complex, biophysical models to experimental data. To test this, we utilized the CoMParE algorithm to fit the L5_AIS model (Spratt *et al*., 2019, 2021), a multi-compartmental L5 pyramidal neuron model that incorporates heterogeneous distributions of sodium channel subtypes (NaV1.2 and NaV1.6) with compartment-specific densities (C. Hu *et al*., 2009), and compared it against the CoMParE-fit BBP model. In Layer 5 pyramidal neurons, the action potential upstroke consists of two distinct components: an initial peak driven by axon initial segment (AIS) sodium channels, followed by a somatic peak (Spratt et al., 2019, 2021; Garcia et al., 2025). The L5_AIS model, which places channels at more physiologically accurate locations, recovers this two-component phase-plane structure, whereas the BBP model does not (Fig. S7). The L5_AIS model has more parameters and hence potentially presents a harder optimization, and is more biologically realistic than models with uniform sodium conductances (NaV1.2, NaV1.6 vs NaT (transient), NaP (persistent)).

**Figure 5.**
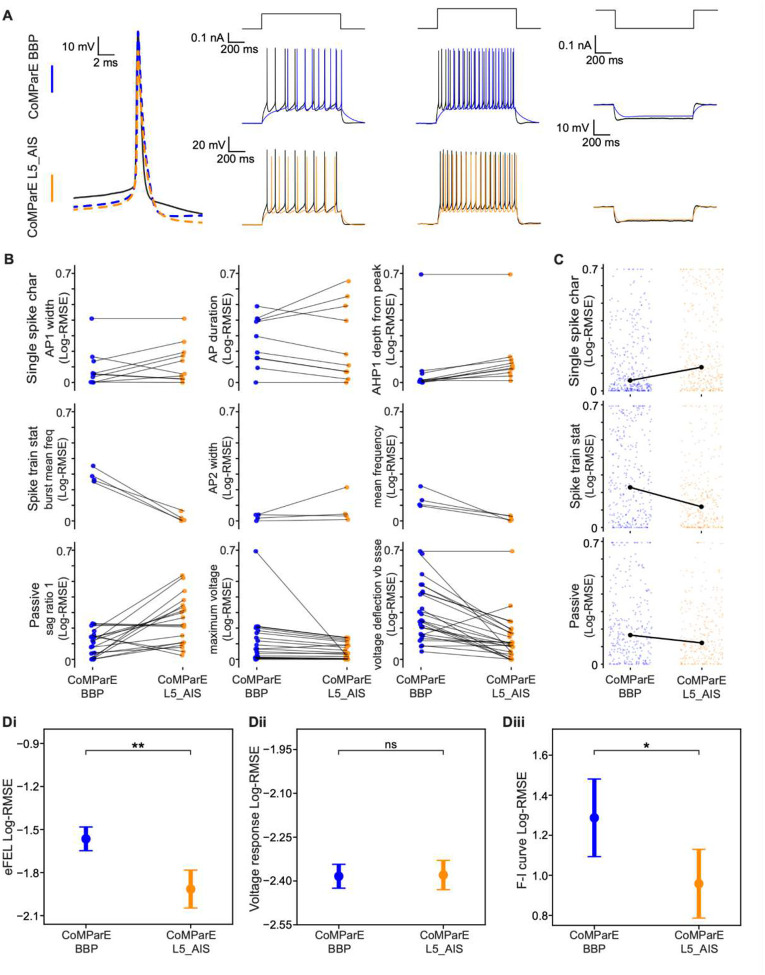
CoMParE enables fitting enhanced biophysically detailed models for improved reproduction of experimental data. A: Example of single (median) spike, spike train and passive responses resulting from three step stimuli for CoMParE BBP from Figure 4 and CoMParE using L5_AIS (CoMParE L5_AIS) model. B: Log root-meansquared-error (log-RMSE) of selected electrophysiological features between a selected experimental cell response and model response for all stimuli. Each dot represents one stimulus, and gray lines connect the same stimulus across the two fits. C: Log-RMSE of all electrophysiological features across all stimuli for a selected cell. Each dot represents one stimulus–feature pair, and black dots connected by lines indicate the median log-RMSE across all pairs for the two fits. Di: Mean log-RMSE of all eFEL+ features across N = 10 fitted cells. Dii: Mean log-RMSE of raw voltage response traces across N = 10 fitted cells. Diii: Mean log-RMSE of frequency-current (F-I) curves across N = 10 fitted cells. Error bars indicate standard error (s.e.). ns, not significant; *p < 0.05, **p < 0.01, Wilcoxon signed-rank test.

Figure 5 illustrates the performance difference between the two models, which use the same evaluation format as Figure 4. The experimental data were fitted with CoMParE, but one with BBP and the other with L5_AIS as the underlying ‘forward’ models (blue and orange, respectively). Figure 5A shows that the L5_AIS model more closely matches the experimental traces (black) than the BBP model, in particular for the spike-train statistics (e.g., smaller first-spike latency errors, more accurate spike counts and interspike intervals (ISIs), middle two), and improved passive response and subthreshold dynamics (middle two and far right). The individual action potential waveforms were largely unchanged (far left). While the voltage traces appear nearly identical, phase-plane analysis (dV/dt vs. V) reveals a critical distinction between the two models (Fig. S7). These observations are quantified in Figure 5B and Figure 5C for this particular experimental data. We found that the BBP model had slightly better quantitative results for single spike characteristics (median log-RMSE, BBP vs. L5_AIS: 0.06 vs. 0.13), but L5_AIS showed better results in spike train statistics (BBP vs. L5_AIS: 0.23 vs. 0.12) and passive features (BBP vs. L5_AIS: 0.17 vs. 0.12). When examined across N = 10 cells, the mean error across all eFEL+ features for L5_AIS was significantly smaller than BBP model’s (L5_AIS vs. BBP: −1.91 ± 0.13 vs. −1.57 ± 0.08, mean ± s.e., p < 0.01, N = 10, WSRT, Fig. 5Di). There was no difference between these models for V_m_ error (L5_AIS vs. BBP: −2.38 ± 0.05 vs. −2.38 ± 0.04, mean ± s.e., p > 0.05, N = 10, WSRT, Fig. 5Dii). Finally, in Figure 5Diii, we found that L5_AIS achieved a better F-I curve error compared to BBP (L5_AIS vs. BBP: 0.96 ± 0.17 vs. 1.29 ± 0.19, mean ± s.e., p < 0.05, N = 10, WSRT). Thus, CoMParE is able to fit enhanced biophysically detailed models that produce V_m_ waveforms that are closer to experimental measurements.

### CoMParE convexifies the objective function landscape through meta-learning

**Figure 6.**
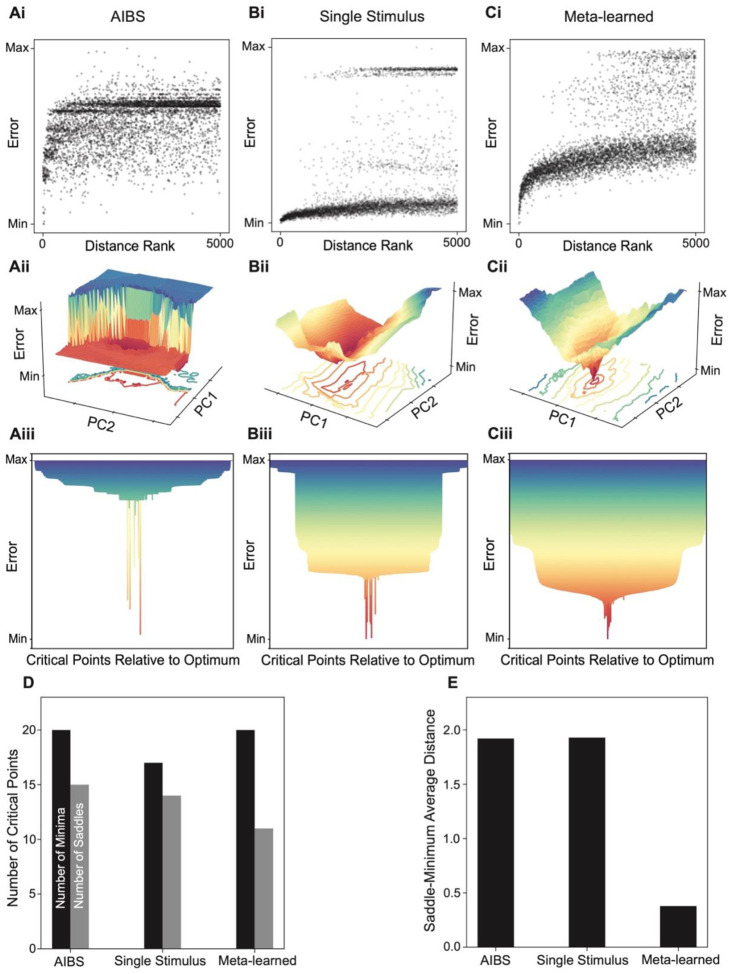
CoMParE convexifies the objective function landscape through meta-learning Ai: Scatter plot of error-rank correlation for AIBS objective function. Aii: PCA visualization of the error surface of AIBS objective function. Aiii: Topological data analysis landscape profiling of AIBS objective function illustrating the critical points near the optimal parameter set. The x axis indicates the relative position of the critical points to the optimum and the y axis indicates the error. Bi: Scatter plot of error-rank correlation for CoMParE-meta-learned objective function using only single-stimulus. Bii: Single-stimulus CoMParE objective function forms a more convex error surface than in Aii. Biii: Single stimulus CoMParE critical points better localize next to optimal parameter sets with lower errors. Ci-iii: CoMParE-meta-learned objective function with full multi-stimuli creates a more convex error surface where critical points are even better localized near the optimal parameter set. D: Quantification of the number of critical points of each objective function. The black bar represents the number of local minima while the grey bar indicates the number of saddle points. The ratio between saddle points and local minima decreases from AIBS to single-stimulus meta-learned to multi-stimuli meta-learned objective function. E: Saddle-minimum average distance (SMAD) quantifies the smoothness of the optimization landscape by measuring average depth of minima. Lower SMAD indicates smoother optimization trajectories toward identifying optimal parameters.

The results above demonstrate that CoMParE meta-learns objective functions that can be optimized to accurately fit parameters of complex biophysically detailed neuron models (Fig. 2–3), enabling them to reproduce experimental data (Fig. 4–5). To gain insight into the underlying structure of the inverse problem, we next sought to quantitatively analyze how the meta-learning approach transforms the optimization landscape. We compared optimization surfaces for three objective functions using the standard BBP model: (1) AIBS objective function constructed in (Gouwens *et al*., 2018) which combines 12 eFEL+ features (Fig. 6A), (2) CoMParE-meta-learned objective function for a single stimulus (Fig. 6B), and (3) CoMParE-meta-learned objective function across multiple stimuli (Fig. 6C). Figure 6Ai–Ci displays scatter plots of parameter distance rank versus error. The AIBS objective function (Fig. 6Ai) exhibits low monotonicity (ρ = 0.42) with substantial scatter, indicating numerous spurious critical points (i.e., parameter values where the objective function is ‘flat’). The single stimulus objective function (Fig. 6Bi) had a greater monotonicity (ρ = 0.70), while the multi-stimulus objective function (Fig. 6Ci) achieved the highest monotonicity (ρ = 0.74). In addition, the multi-stimulus approach exhibited a narrower spread of distance rank around the global minimum (lower-left corner of the plot), suggesting a more sharply sloped function as we approach the optimum, and thus allowing model parameters to be fit more accurately and robustly.

To evaluate the ‘convexity’ of the objective function landscape we systematically varied model parameters to numerically quantify the gradient of the different objective functions with respect to the parameters (Methods). We first visualized the geometry of the objective function landscape using PCA to extract the low-dimensional subspace that captured the most variance in the gradient of the objective function across features (Fig.6 Aii–Cii, see Methods). The AIBS objective function (Fig. 6Ai) had several extensive flat regions, indicating many different sets of parameter values are indistinguishable through the lens of optimization algorithms evaluated on this objective. The single-stimulus surface (Fig. 6Bii) no longer had broad flat regions, but exhibited regions with relatively shallow slopes, especially around the global minima. The multi-stimulus objective function meta-learned by CoMParE (Fig. 6Cii) had high slopes and a sharply defined global minimum, indicating that the CoMParE meta-learned objective function is more favorable for optimization.

We next used topological data analysis to directly identify critical points (minima and saddles) across the entire landscape (see Methods). Figure 6Aiii–Ciii plots the value of the objective function (y-axis) for all identified critical points (the x-axis indexes the identified critical points ordered relative to their distance from the global minima in the middle of the x-axis). The AIBS approach had distantly separated minima (downward spikes separated near the middle of the x-axis) and a proliferation of critical points with similar high error (multiple plateaus), indicative of gradient flat regions or saddles (Frye *et al*., 2021). The single-stimulus approach had smaller separation across the minima and fewer high-value critical-points, but still many critical points with distinct values, again indicative of many saddles. The CoMParE meta-learned objective function had several minima (clustered downward spikes) close to the global minima, few critical points with high error, and very few distinct error values, indicative of a reduction in the number of saddles. Figure 6D summarizes the critical point analysis. The AIBS approach had 20 minima and 15 saddle points, single-stimulus meta-learning resulted in 17 minima and 14 saddles, while multi-stimulus meta-learning resulted in 20 minima and 11 saddle points. Finally, to quantify the average “depth” of minima in the landscape, Figure 6E shows the Saddle-Minimum Average Distance (SMAD) metric (lower SMAD values indicate shallower minima and hence a smoother landscape that is easier to optimize, see Methods). The progression from the AIBS objective function (SMAD = 1.92) to multi-stimulus meta-learned objective function (SMAD = 0.38) represents a 5-fold improvement in landscape smoothness. Taken together, these results indicate that CoMParE meta-learned objective functions remove saddles and cluster minima around the global minima, transforming the optimization landscapes into more convex surfaces that are amenable to accurate and reliable parameter fitting.

## Discussion

Determining the precise biophysical properties of neurons that govern membrane potential dynamics is fundamental to understanding the brain in health and disease. For example, it could provide a direct bridge from experimentally accessible voltage dynamics to the channel-level mechanisms that implement neural computations (Koch, 1998), enable tracking how conductance profiles mature through activity-dependent mechanisms during development (Moody and Bosma, 2005), and clarify how channelopathy mutations alter specific conductances to produce pathological dynamics in disease (Kullmann, 2002; Happ and Carvill, 2020). Despite this promise, current experimental and computational approaches have not yet delivered a robust capability to determine conductances from membrane potential recordings. Here, we show that the combination of meta-learning objective functions across diverse stimuli and improved biophysically detailed models can result in more accurate and precise fits of model parameters and improved reproduction of experimental V_m_ data.

### Addressing the inverse problem of single-neuron membrane potentials.

Many inverse problems in neuroscience are mathematically degenerate (i.e., many distinct combinations of sources produce the same measurement), such as identifying the precise neuronal sources that combine to produce LFP, ECoG, or EEG. The work of Marder and colleagues further demonstrated that disparate conductance configurations can result in similar circuit-level rhythmic output (Goldman *et al*., 2001; Prinz, Bucher and Marder, 2004). However, whether or not a diverse constellation of conductances give rise to identical membrane potentials is obscured by methodological issues. Experimental electrophysiology methods alone cannot fully capture the functional ‘snapshot’ of ion channels active when a neuron’s behavior is recorded: voltage-clamp requires pharmacological isolation of individual currents across extended protocols, while current-clamp captures only the aggregate voltage without directly revealing underlying conductances (Golowasch et al., 2002; Bano-Otalora et al., 2021). Computational approaches have attempted to fill this gap by fitting biophysically detailed neuron model conductances to current-clamp recordings, which do capture the natural interactions of all ionic currents within a cell (Keren, Bar-Yehuda and Korngreen, 2009). Fitting detailed models to experimental data has been a major challenge for decades (Bhalla and Bower, 1993; Vanier and Bower, 1999; Prinz, Bucher and Marder, 2004). However, many fitting pipelines rely on *ad hoc* objective functions built from manually selected electrophysiological features (Druckmann *et al*., 2007; Gouwens *et al*., 2018; Ladd *et al*., 2022). Thus, no prior work directly demonstrates that the degeneracy encountered in practical conductance fitting of single neuron models is an intrinsic property of neuronal biophysics or is partly induced by the methodology used.

To address this gap, we developed CoMParE (Conductance-based Model Parameter Evaluation) with the goal of enabling determination of ionic conductances from membrane potential recordings. In synthetic data, we demonstrate that CoMParE achieved state of the art performance in accurately fitting parameters of biophysically detailed neuron models (Fig. 3). We found that not all parameters were fit with equal fidelity–parameters associated with action potentials were fit most accurately (low mean error) and precisely (low variance of error). Generally, parameter fitting accuracy and precision were strongly correlated, indicating that some parameters are ‘tight’ while others are ‘sloppy’ with respect to the *somatic* V_m_ recordings. While we focus on somatic recordings, as those are the most experimentally prevalent, we propose that multi-site V_m_ recordings, such as simultaneous somatic and dendritic/axonal recordings, would constrain compartment relevant conductances that were poorly fit to somatic V_m_, and enable accurate and precise fitting of them. Indeed, we demonstrate that CoMParE achieved substantially better reproduction of experimental membrane potential dynamics compared to the state-of-the-art approach (Fig. 4). Together, these results indicate that the inverse problem of single-neuron membrane potentials may not be fundamentally degenerate. Instead, we demonstrate that improved computational and experimental approaches can be used to determine the values of conductances from intracellular membrane potential recordings.

### Meta-learning across diverse stimuli reduces optimization-induced degeneracy.

In practice, because fitting single-neuron models to V_m_ is currently formulated as a multi-objective optimization problem, most methods utilize linear scalarization by averaging the error across multiple (weighted) features of V_m_. However, fitted parameters can be sensitive to the weight choice, creating inconsistent estimates and an appearance of degeneracy (Das and Dennis, 1997; Marler and Arora, 2010). Traditional neuron-model fitting approaches utilize objective functions constructed out of manually selected electrophysiological features with *ad hoc* weighting schemes (Druckmann *et al*., 2007; Gouwens *et al*., 2018). When error reduction is poorly aligned with proximity to the correct parameters, such objective functions can create rugged optimization landscapes with minima and ‘gradient flat’ regions (i.e., saddles) that are far from the global optimum. This can result in fit parameters that deviate substantially from the ‘true’ values. CoMParE addresses this by meta-learning objective functions (weights on V_m_ features) that maximize the Spearman rank-correlation (ρ) between error and (rank-) distance from ground-truth parameters. High ρ indicates that reductions in error are consistently aligned with movement toward the ground-truth region in parameter space, so descent in the objective tends to correspond to progress toward the correct parameters. To the best of our knowledge, our use of Spearman Rho to identify feature weightings that maximize monotonicity between feature distance and error is a novel approach in multi-objective optimization. We note that methods for direct analysis of high-dimensional objective function landscapes are typically computationally challenging and scale poorly with dimensionality (Gyulassy *et al*., 2008; Gerber *et al*., 2010; Otter *et al*., 2017). Instead of directly identifying and attacking local critical points (Dauphin *et al*., 2014), CoMParE combines meta-learning with biophysically detailed simulations across diverse stimuli to modify the objective function to return more accurate fits. Nonetheless, our geometric and topological analysis of objective function landscapes revealed that CoMParE meta-learning systematically removes deleterious critical points (i.e., minima and gradient flat regions distant from the global optima) and clusters minima near globally optimal values (Fig. 6) so that optimization over this landscape is more likely to result in high-quality fits.

The neuron is a complex, electrophysiological system with multiple parameters that interact in non-trivial ways to produce observed membrane potential dynamics. CoMParE also leverages the idea that model fitting becomes more robust and generalizable when the neuron is probed under diverse conditions. When fitting is performed under a stimulus set that engages a narrow set of parameters (e.g., step, ramp), many parameterizations can appear equivalent because they only need to reproduce a limited slice of the neuron’s dynamics. Such stimulation protocols can be very insightful for experimental interrogation of specific conductances. However, from a system-identification perspective, more dynamic inputs such as the ‘chaotic’ and ‘chirp’ stimuli probe a broader range of dynamical regimes and therefore can expose more information about the underlying parameters than simple ‘step’ or ‘ramp’ stimuli (Druckmann *et al*., 2011). CoMParE builds on this principle by meta-learning objective functions across diverse stimulation conditions, effectively turning “many solutions fit one condition” into “fewer solutions generalize across conditions.” Most cellular neurophysiology data is currently collected using the canonical simple stimuli, yet our results advocate for a more diverse and dynamic set of stimuli to be used to enable the complete identification of the electrophysiological system generating membrane potentials. Because we focused on layer 5 pyramidal cells, whether this advantage extends similarly to neuronal classes with different morphologies and channel complements remains to be determined.

### Robust fitting algorithms and biophysically detailed models reinforce each other.

A common assumption in computational neuroscience is that simpler models are preferable because they have fewer parameters, and thus less prone to overfitting. This reasoning has driven skepticism about fitting highly detailed biophysical models to experimental data and promoted the widespread use of reduced models (Izhikevich, 2004; Burkitt, 2006; .Paninski and Cunningham, 2018). Our results directly challenge this concept. Combined with CoMParE, the L5_AIS model (Spratt *et al*., 2021; Garcia *et al*., 2025), which incorporates additional details of the heterogeneous distributions of sodium channel subtypes, resulted in improved reproduction of experimental data compared to the simpler BBP model (Fig. 5). The improvement was particularly evident in spike train statistics and firing rate-current relationships. This demonstrates that the additional biological detail simultaneously provides crucial degrees of freedom and constraints that simpler models do not. Notably, the consistent accuracy achieved across N = 10 individual neurons — using a single shared morphological template rather than cell-specific reconstructions — suggests that CoMParE generalizes across the natural morphological variability present within a cell type, reducing a significant practical barrier to routine model fitting (Figs. 4, 6).

Together, our results demonstrate that a substantial contributor to the prior difficulty of fitting biophysically detailed models reflects insufficiencies in the optimization methods, rather than the over-parameterization of the model *per se*. Recent data assimilation work also suggests that over-specification alone does not explain degenerate parameter estimates (Nogaret, 2022; Wells *et al*., 2024). That is, additional biological detail can become an asset by increasing model expressivity and implicitly enforcing constraints. Robust fitting algorithms and detailed models can therefore reinforce each other, enabling more accurate and precise determination of ionic conductances from membrane potential recordings.

The identifiability of parameters in electrotonically distant compartments remains fundamentally limited by the information content of somatic recordings. Combining CoMParE with multi-site recording techniques, voltage imaging, extracellular recordings, or other modalities that provide spatial information about dendritic and axonal activity may therefore further improve parameter identifiability (Keren, Peled and Korngreen, 2005; Antic *et al*., 2010). Thus, our results advocate for re-invigoration of the ‘virtuous cycle’ between experiments and modeling in cellular neuroscience.

## Methods

### Cell models

The Hodgkin-Huxley (HH) model and the Blue Brain Project (BBP) Layer 5 thick-tufted pyramidal cell model (Ramaswamy *et al*., 2015) were used for theoretical experiments to demonstrate the proof of concept to validate CoMParE’s ability to recover known ground-truth conductance parameters from simulated voltage data (HH model, Fig. 2; BBP model, Fig. 3). For fitting to experimental data (Fig. 4–5), the BBP model and L5_AIS model (Spratt *et al*., 2021) were used to demonstrate the impact of model complexity on the CoMParE algorithm. Neuronal membrane potential dynamics were modeled using conductance-based formalisms, wherein voltage-gated ion channels are characterized by maximal conductance parameters governing transmembrane currents.

#### Hodgkin-Huxley model (Table S2):

A single-compartment Hodgkin-Huxley model was used for the experiments in Figure 2. The model consisted of a spherical soma (L = 15 μm) containing the canonical HH conductances: fast sodium (gnabar_hh), delayed rectifier potassium (gkbar_hh), and passive leak (gl_hh). This minimal 3-parameter model provides a tractable test case for validating objective function optimization, as the reduced parameter space permits exhaustive characterization of the fitness landscape. Resting potential was initialized at −65 mV. Parameters were bounded at [0.01×base, 100×base] for optimization.

#### BBP model (Table S3):

The Blue Brain Project thick-tufted layer 5 pyramidal cell model (*cADpyr232*) was used for the validation experiments (Fig. 3). This model contains numerous biophysical parameters, of which 24 (comprising maximal ionic conductance densities and membrane properties) were treated as free parameters for optimization, distributed across axonal, somatic, apical, and basal compartments. Axonal compartments were populated with transient sodium (*NaTa_t*), persistent sodium (*Nap_Et2*), delayed rectifier potassium (*SKv3_1*), transient potassium (K_Tst), persistent potassium (*K_Pst*), calcium-activated potassium (*SK_E2*), and high- and low-voltage activated calcium (*Ca_HVA*, *Ca_LVAst*) conductances. Somatic compartments contained transient sodium (*NaTs2_t*), delayed rectifier potassium (*SKv3_1*), calcium-activated potassium (*SK_E2*), both calcium channel types, and hyperpolarization-activated cation current (*Ih*). Apical dendrites were assigned transient sodium, delayed rectifier potassium, muscarinic potassium (*Im*), and Ih with distance-dependent distribution. Basal dendrites contained only Ih. Intracellular calcium handling was governed by calcium dynamics parameters (gamma, decay). A uniform passive leak conductance (*g_pas*) was applied throughout all compartments, with reversal potential (*e_pas* = −75 mV) and membrane capacitance (*cm* = 1 μF/cm^2^ somatic, 2 μF/cm^2^ dendritic) held fixed at default values. All optimizable conductance parameters were bounded at [0.01×base, 100×base], spanning four orders of magnitude.

#### CoMParE BBP model (Table S4):

A reduced 16-parameter configuration of the BBP model was constructed for comparative analyses in Figure 4. Apical active conductances (*NaTs2_t*, *SKv3_1*, *Im*), calcium-activated potassium (*SK_E2*), somatic Ih, and calcium dynamics parameters were fixed at their default biophysical values. Passive membrane properties (*e_pas*, *cm*) were added as free parameters to enable optimization of resting potential and membrane time constant. Dendritic Ih was unified across basal and apical compartments into a single optimizable parameter (*gIh_dend*). This parameterization reduces optimization complexity while preserving the ion channel complement responsible for action potential generation and spike frequency adaptation.

#### L5_AIS model (Table S5):

A layer 5 thick-tufted pyramidal cell model with explicit axon initial segment (AIS) representation was adapted from Spratt et al. (2021) (Spratt *et al*., 2021) to examine the effect of increased model complexity on CoMParE performance (Fig. 5). This model, originally developed to investigate paradoxical hyperexcitability arising from NaV1.2 sodium channel loss in neocortical pyramidal cells, distinguishes between Nav1.2 (*SCN2A*) and Nav1.6 (*SCN8A*) sodium channel subtypes. Following the BBP L5 pyramidal cell framework, the reconstructed axon was replaced with a simplified axon stub. The proximal axon section was set to 90 μm and discretized into 19 segments to accommodate an AIS. Nav1.2 and Nav1.6 conductances were each split into two “alleles” (*na12/na12mut* and *na16/na16mut*) to enable flexible modeling of channel mutations. Conductances were distributed between the paired alleles such that their sum matched the intended total channel density to preserve total gNa while enabling allele-specific perturbations. In the work detailed in this manuscript, the same mechanism parameters were inserted in both alleles of each respective channel to simulate a wildtype neuron, with the allele split primarily serving as scaffolding for future experiments. Nav1.2 and Nav1.6 channels were distributed across dendritic, somatic, and axonal compartments, reflecting experimentally observed differential expression along the somato-axonal axis that governs action potential initiation and backpropagation. The 90 μm length of the first axon section was divided to create the AIS in the first 52 μm proximal to the soma. The 19 subdivisions creating the AIS contain Nav1.2 and Nav1.6 differentially inserted to mimic distributions obtained from experimental results (W. Hu *et al*., 2009). Nav1.2 is elevated proximally, peaking at 10.5 μm from the soma and decreasing to zero near the middle of the AIS (21 μm from soma). Nav1.6 increases from the beginning of the AIS, peaking at 21 μm then remains constant until the end of the AIS (52 μm). In addition to fast spiking currents, baseline axonal excitability was further shaped by inclusion of calcium-activated potassium channels in the axonal compartment to support more flexible regulation of spike initiation alongside sodium channel redistribution.

The model comprises 26 optimizable parameters including compartment-specific passive properties (gpas, cm, e_pas specified independently for soma, dendrites, and axon), multiple potassium conductances (*K, KP, KT, KCa*), high- and low-voltage activated calcium (*HVA*, *LVA*), nodal sodium (*node_na*), and dendritic Ih.

##### Conductance-based Model Parameter Evaluation (CoMParE) algorithm

###### Overview.

The CoMParE meta-learning algorithm systematically explores the model parameter space to learn an objective function ($h:R^{N}\to R$, where *N* is the number of model parameters) that linearly combines eFEL+ features. For a given neuron model, this requires evaluation of ρ(Err, DR) with respect to a broad set of model parameters to map the transformation of a diverse set of current injection (I_in_) stimuli to evoked V_m_, as well as evaluation of diverse features to calculate Err. To do this, the core procedure of the CoMParE algorithm is broken down into five steps. (1) For a given neuron model (e.g., Hodgkin-Huxley) and a defined ‘target’ set of model parameters (M*), CoMParE generates a diverse collection of model parameter sets (M’, e.g., variations in conductances, ~1000 variants). Thus distance rank (DR) is defined as a ranking of smallest to largest euclidean distance between all M’ and M*. (see Parameter space exploration section below). (2) CoMParE then simulates V_m_ across the different M’ (producing V_m_^M’^) and V_m_ for the target parameters (producing V_m_^M*^) for a diverse set of I_in_ (e.g., variants of ramp, chirp, chaotic, step, see Choice and generation of stimuli below). (3) The difference (Err) between V_m_^M*^ and V_m_^M’^ is calculated using a broad set of canonical electrophysiological evaluation features (eFEL+, e.g., Time to first spike, Mean action potential amplitude, see Linear combination of electrophysiological (eFEL+) features section below). (4) The final CoMParE objective is optimized by linearly combining the weights (ŵ) of canonical electrophysiological evaluation features that maximize ρ(Err, DR) (see Error function weight optimization section below). (5) With the finalized CoMParE objective, neuronal model parameters are fitted with EA algorithms (for specific implementation, see Evolutionary algorithm implementation section below). The pseudocode for full CoMParE algorithm is provided in Supplementary Pseudocode.

###### Parameter space exploration.

Exploration of parameter space is a critical step to selecting the best combination of eFEL+ features and stimuli by examining change of voltage response with respect to deviation of conductance parameters (M’) from the ground truth (M*). To pick a reasonable starting point, the original model (e.g., Hodgkin-Huxley) parameter is selected as a proxy for the ground truth conductance parameter set. With respect to ground truth values, the minimum and maximum range of exploration is chosen as [0.01*p*_*i*_, 100*p*_*i*_] for each ground truth parameter *p*_*i*_. Because these parameters span a range that differs in magnitudes, they are first sampled from a normalized hypercube of *N* dimensions ([0, 1]^*N*^), where *N* is the number of conductance parameters of the neuronal model of interest. For efficient sampling, each axis of the *N*-dimensional hypercube [0, 1]^*N*^ is partitioned into *k* (in all of our experiments, we fixed *k* = 10 but this is a tunable parameter) equal subintervals: $\left[0,\frac{1}{k}\right),\left[\frac{1}{k},\frac{2}{k}\right),\cdots ,\left[\frac{k-1}{k},1\right]$. Each resulting sub-hypercube, defined by the Cartesian product of these intervals across all dimensions, is then uniformly sampled with the same number of samples per sub-hypercube. While the number of samples is a tunable parameter, we sampled 1000 parameter sets when using the HH model (Fig. 2) and 5000 parameter sets for all other experiments. After sampling, each sample is mapped to the correct range using linear mapping.

###### Linear combination of electrophysiological (eFEL+) features.

The functions in eFEL+ are used to extract features from voltage response of a neuronal model stimulated with a stimulation protocol (Fig. S1). Using these features, a function that measures the deviation of a voltage response from the ground truth with respect to those features is constructed by computing the l_2_ distance between the features. Since different eFEL+ functions will measure various aspects of a voltage response, some are more important than others while some are redundant or irrelevant. To select the best combination of these features, an optimization algorithm was used to determine a linear combination of relevant functions (see Error function weight optimization below for specific details). In addition to published Electrophys Feature Extraction Library (eFEL, (Ranjan *et al*., 2024)) features, a small number of custom features are also utilized. For simplicity the whole set of features are referred to as ‘eFEL+’ throughout the manuscript. The complete list of eFEL+ features considered are listed in Table S1.

Even with the same eFEL+ function, the functional behavior is significantly different when the neuron is stimulated with a different stimulation protocol. Therefore, an eFEL+ function paired with a stimulus is treated as one error function in construction of optimization problems. Specifically, for *m* eFEL+ features *f*_1_, *f*_2_, …, *f*_*m*_ and *n* stimulus *s*_1_, *s*_2_,…, *s*_*n*_ an ‘elementary’ error function is described as *g*_*i,j*_ = (*f*_*i*_, *s*_*j*_) and the final objective function have form (see Error function weight optimization below for more details on how the objective function is constructed)

(1) $$h=\sum_{i,j}\;w_{i,j}g_{i,j}$$

Note that if the neuronal model is fixed, the voltage response for a given stimulus *s* will only depend on the conductance parameter *p* of the model. Since an elementary error function *g*_*i*,*j*_ measures deviation in voltage response from the ground truth with respect to specific stimulus *s*_*j*_ and eFEL+ feature *f*_*i*_, it is a function of the parameter set *p*. Thus, *g*_*i,j*_ is a function of *p*, resulting *h* to be a function of *p* and the weight vector *w*.

###### Error function weight optimization.

‘Elementary’ error functions (*g*) will differ in importance according to the neuronal model and the voltage response gained by the experiments (ground truth). To incorporate such relevance, weights are assigned using optimization algorithms for each elementary error function. This optimization problem is constructed by maximizing the Pearson’s correlation (*r*) between the rank of conductance parameter sorted by l_2_ distance from original BBP parameter *p*^*o*^ and the rank of the error function evaluated with *w*. Concretely, for conductance parameter *p* = [*p*_1_, *p*_2_,…, *p*_*n*_]^*T*^ and error function *h*(*p*, *w*), the weights are optimized by the following

(2) $$w^{*}={\text{argmax}}_{w}\;r\left(\text{rank}\left({\left\|p-p^{o}\right\|}_{2}\right),\text{rank}\left({\left\|h\left(p,w\right)-h\left(p^{o},w\right)\right\|}_{2}\right)\right)\;\text{for}\;\text{all}\;p,\;\text{s.t.}\;w_{i}\in \left[0,1\right].$$

Note that *h*(*p*^*o*^,*w*) = 0 because *h* measures voltage response deviation from *p*^*o*^ but addressed in the equation for completeness.

For the optimization of (2), we deployed a stochastic optimization that is robust to noise (pattern search algorithm, (Mayer *et al*., 2016). Also, because each error function spans a wide range of numerical values, they are normalized to the range of [0, 1] using the minmax normalization method before the weights are assigned.

Out of 300 stimuli that were generated, the best 20 are selected to construct the multi-stimuli meta-learned error function (see Choice and generation of stimuli below for more details). To choose the best 20 stimuli, error function optimization problem is solved for every 300 stimuli individually, computing the corresponding maximum Spearman’s correlation coefficient. According to the maximum Spearman’s correlation coefficient, the top 20 stimulus is chosen. With the top 20 stimuli, the optimal weights are recomputed as the final form of the meta-learned error function.

###### Choice and generation of stimuli.

In addition to classic stimulation protocols (such as impulse, step, ramp, and noise), we included chaotic stimuli (Meliza *et al*., 2014), chirp, and sine waves. The amplitude and frequency range was between [3, 9] nA and [0, 280] Hz, respectively for chirp stimuli and [3, 9] nA and [100, 1000] Hz for sine wave stimuli. These parameters were chosen to sample a range of membrane potential fluctuations, subthreshold oscillations, action potential firing patterns, and depolarization blocks. We validated that these stimuli lied within physiological range and produced robust, consistent responses by applying these stimuli in slice whole-cell current clamp recordings of layer 5 cortical pyramidal neurons in mouse prefrontal cortex (data not included here).

###### Evolutionary algorithm implementation.

The optimization problem considered in this paper is the fitting of biophysically accurate parameters of a neuron using evolutionary algorithms (EAs). The following description follows from previous work (Ladd *et al*., 2022). EAs are a class of optimization methods that rely on natural selection in a population through biologically inspired operators such as mutation, crossover, and fitness-based selection (Mitchell, 1998). Solutions to the optimization problem were encoded as continuous vector representations of neuron model parameters. We refer to this group of parameterized neuron models as the “population” and a single model as an “individual”. The quality of each individual was assessed by evaluating the CoMParE-meta-learned objective function, which compares model responses to target electrophysiological data. The algorithm employed was the (μ+λ) evolutionary algorithm (Beyer and Schwefel, 2002) and was implemented using DEAP (De Rainville *et al*., 2014) and BluePyOpt (Van Geit *et al*., 2016). μ and λ denote the size of the parent population and the number of offspring to produce for the next generation, respectively. In this implementation, μ denotes the size of the parent population and λ the number of offspring produced per generation. Offspring were generated via simulated binary crossover (SBX) with η = 20 and polynomial mutation with η = 20, applied with crossover probability = 0.7 and mutation probability = 0.3, respectively. Parent selection was performed using the Indicator-Based Evolutionary Algorithm (IBEA) with indicator-value tournament selection (Zitzler and Künzli, 2004).

The EA proceeds as follows (see Supplemental Pseudocode):

1. Initialization. A parent population of μ individuals was initialized by uniformly sampling parameter values within the specified bounds.
2. Evaluation. Each parent individual was evaluated by simulating the neuron model with its parameter set and computing the weighted-sum objective score against target electrophysiological data.
3. Variation. For each generation, λ offspring were produced from the parent population via the VARIATION operator, which applied mutation, reproduction, or crossover exclusively to each individual (or pair, in the case of crossover) according to the specified crossover and mutation probabilities.
4. Offspring evaluation. Each offspring individual was evaluated using the same objective function.
5. Selection. The parent and offspring populations were combined, and μ individuals were selected to form the next parent generation using IBEA tournament selection.
6. Hall of fame update. A hall of fame tracking the 10 lowest-scoring individuals encountered across all generations was updated.
7. Iteration. Steps 3–6 were repeated for nGenerations.
8. Termination. Upon completion, the individual from the hall of fame with the lowest objective score was returned as the optimal parameter set.

###### Generalization.

To test the generalization capacity of multiple-stimulus meta-learned error functions, it is tested to recover different parameter sets that are randomly generated. The parameter to recover is generated from $\left[\frac{p^{o}}{5},5p^{o}\right]$ where *p*^*o*^ is the original BBP parameters. In addition, the voltage response derived from the chosen parameter set is visually inspected to ensure that it produces biophysically plausible voltage response. With the generated surrogate parameter set *p*^*s*^ and corresponding voltage response, the CoMParE algorithm was tested with this voltage response as the optimization target. During this test, no additional modification was done to the meta-learned error function.

As an evaluation metric, deviation from the new ground truth parameter *p*^*s*^ was measured for each parameter optimized (see Evaluation of fit accuracy via parameter distance from ground truth section below).

### Fit Parameter Evaluation

#### Evaluation of fit accuracy via parameter distance from ground truth.

The evaluation of distance between fitted model and new ground truth parameters was measured as

(3) $$D_{i}=\left|\text{log}\left(p_{i}/{p^{*}}_{i}\right)\right|$$

where *p*_i_* are the known ground truths and *p*_*i*_ are the estimated values for parameter *i*.

With each *D*_*i*_, the overall distance is computed as the mean across all parameters, which describes log distance from the target metric in Figure 2H and Figure 3J.

#### Evaluation of parameter fit robustness.

In Figure 3K, parameter fit robustness across N = 20 fitting experiments with different EA random seeds is examined. The random seed of EA determines the starting points in the parameter space for every offspring. To test if a conductance parameter can be robustly recovered regardless of the different starting points, variance of error is compared to the mean of error for every parameter across N = 20 seeds (for details on error, see Evaluation of fit accuracy via parameter distance from ground truth above). To quantify the correlation between mean error and variance of error, coefficient of determination (R^2^) is computed. In addition, a simple linear regression is performed and the result is reported with 95% confidence interval as shown in the Figure 3K.

#### eFEL+ feature categorization.

The eFEL+ features are categorized into four categories of single spike characterization, spike train statistics,passive and general. The categorization is derived from the documentation of the eFEL with some modifications. The full categorization is described in Table S1. Example eFEL+ features are illustrated in Fig. S1.

### Fitting experimental data

To fit experimental data, we used the following procedure:

1. We ingested experimental neural data from https://celltypes.brain-map.org/data as an NWB file and then downsampled stimulus duration to a uniform 30000 timesteps. These downsampled stimuli and experimental voltages were saved in HDF5 files as HDF5 file format offered memory and read efficient storage. The new dt was saved as well.
  - Cells were chosen as follows. We filtered for mouse neurons with all-active biophysical models and the following transgenic lines: RBP-Cre, RORB-IRES-Cre, Nr5a1-Cre, Scnn1a-Tg3-Cre, Gng7-Cre, and Cux2-Cre.
2. We then sampled 5000 parameter sets according to the Parameter space exploration section above.
3. We then simulated responses for 3–5 long step suprathreshold sweeps (stimuli), 2 long step subthreshold sweeps, and 2 short step sub/suprathreshold sweeps.
4. We then scored the simulations using eFEL+ error functions.
5. This enabled us to generate the multi-stimulus objective function for EA following the Error function weight optimization section above.
6. We then ran EA for each model for at least 40 generations of population size 5000. In all cases, EA was run until the score stabilized (< 1% change per generation). We then selected the individual parameter set that minimized the CoMParE meta-learned objective function as the final result of EA optimization.
7. We then used the best model from step 6 to generate responses for all original (non-downsampled) sweeps in the Allen Institute data. This resulted in waveforms comparable to the waveforms generated by the (Allen Institute for Brain Science, 2015) for the open access Allen Institute models.

### Implementation

The computational framework was deployed on NERSC’s Perlmutter supercomputer. A single bash script orchestrated job submissions with dependencies managed through SLURM scheduling. Each optimization job utilized batch scripts that executed Python code in parallel across all allocated CPU cores using MPI4PY 4.1.0. Individual evolutionary algorithm optimizations ran for 6 hours on 4 nodes (256 total CPU cores, 64 cores per node on AMD Milan processors). The software stack consisted of: Cray MPICH 8.1.30 for MPI communication, Python within a dedicated virtual environment, and the GNU Programming Environment 8.5.0. The evolutionary algorithm was implemented using DEAP 1.4.3 (De Rainville *et al*., 2014) and BluePyOpt 1.14.18 (Van Geit *et al*., 2016). Fitness evaluations were computed using the Blue Brain Project’s Electrophys Feature Extraction Library (eFEL) 4.3.0 (Van Geit *et al*., 2016). Note that the list eFEL features changes in newer releases. AllenSDK 2.16.2 was used to run Allen Institute models and prepare open-source electrophysiology data. Biophysical neuron simulations were executed using NEURON 8.2.2 with compiled .mod files accessed through NEURON’s Python interface.

Core Simulation & Optimization Tools:

- NEURON: https://github.com/neuronsimulator/nrn
- BluePyOpt: https://github.com/BlueBrain/BluePyOpt
- DEAP (Distributed Evolutionary Algorithms in Python): https://github.com/DEAP/deap

Feature Extraction & Analysis:

- eFEL (Electrophys Feature Extraction Library): https://github.com/BlueBrain/eFEL
- AllenSDK: https://github.com/AllenInstitute/AllenSDK
- IPFX (Intrinsic Physiology Feature Extractor): https://github.com/AllenInstitute/ipfx

Additional Relevant Tools:

- MPI4PY: https://github.com/mpi4py/mpi4py
- Allen Brain Cell Types Database: https://celltypes.brain-map.org/data

### Analysis of Objective Function

#### Visualization and analysis of CoMParE objective function via PCA.

In order to more comprehensively examine the effect of CoMParE-meta-learned objective functions, we observed how the objective function behaves when stepped in maximally varying directions. We found two most varying directions in parameter space by taking principle component analysis on the gradient of objective functions: Let $\;f:R^{n}\to R$ be an objective function, we define gradient function *g* = *∇f* and take the top 2 principal components *p*_1_, *p*_2_ of function *g*. We then evaluate *f* as a function of *p*_1_, *p*_2_ to examine most dominant local minimas.

#### Topological Data Analysis.

To characterize the meta-learned objective function, we employ the topological landscape profiling method introduced by (Oesterling *et al*., 2013), which uses merge trees to identify and represent critical topological features, such as minima, saddle points, and their connectivity, in high-dimensional functions. In our application, the objective function is evaluated over a two-dimensional meshgrid constructed around the ground truth parameter vector from the BBP model. This meshgrid spans the most informative subspace of the parameter space, allowing us to effectively capture the landscape’s essential structure. By treating low-score regions as valleys rather than high-score regions as hills, we reinterpret the merge tree metaphor to reflect optimization rather than clustering: valleys correspond to minima, basins represent stable regions, and connecting saddles indicate potential escape paths between them. The resulting topological landscape profile provides an interpretable summary of the objective function’s geometry, revealing dominant minima and the transitions between them in a compact and visually intuitive form.

#### Objective function behavior characterization.

To quantitatively assess the sharpness and separation of the basins in this landscape, we introduce the Saddle-Minimum Average Distance (SMAD). Formally, SMAD is defined as:

(4) $$\text{SMAD}=\frac{1}{m}\sum_{i=1}^{M}\;\left|ps_{i}-pm_{i}\right|$$

where *M* is the number of branches in the merge tree extracted from the landscape. For each branch *i*, *ps*_*i*_ denotes the persistence value of the saddle point—representing the difference in score between the saddle and the associated minimum—while *pm*_*i*_ denotes the persistence of the minimum itself. Persistence, a central concept in persistent homology, reflects how long a topological feature (such as a basin) exists as the function is continuously filtered. SMAD therefore captures the average “depth” of the basins in the landscape. A lower SMAD indicates shallower, more homogeneous basins and a smoother landscape, whereas a higher SMAD suggests deeper or more divergent basins with pronounced sharpness or separation, which manifest as longer branches in the merge tree.

## Supplementary Files

This is a list of supplementary files associated with this preprint. Click to download.

- OptimizationPaperSupplementary.docx

## Data Availability

Code for the pipeline can be found at: https://github.com/xanderladd/axonstandardized Data for fitted models and runs can be found at: https://portal.nersc.gov/cfs/m2043/CoMParE Notebooks/Visualization can be found at: https://github.com/xanderladd/CoMParE_figures

## Technical Box 1. Meta-learning Objective Functions

Intuitively, if the ground-truth relationship between a parameter (e.g., and produced V_m_ is unique, then a ‘good’ objective function should have a ‘simple’ relationship between its values and the difference between a model’s parameters and the ground truth parameters. Formal metrics of the geometry of objective function landscapes (e.g., negative eigenvalues of Hessian) are often computationally intensive. Thus, while the goal is to find this ‘good’ objective function, the challenge is to devise a simple metric of the ‘quality’ of an objective function for optimization that can be used in creating the ‘good’ objective. To build intuition, Panel Ai shows an example where the ‘error’ (z) is a nonconvex function of two parameters (x & y, see Methods). This function has many critical points (i.e., [x, y] pairs for which small changes in x or y yield approximately the same error): a global minimum at [0, 0], a pronounced local minimum at [0, 1], and a ~saddle-point (gradient flat regions) at [1, −1]. These characteristics would challenge an optimizer to find the global minimum (e.g., the true values of model parameters), as the optimizer could get stuck at the non-global critical points. This function exhibits only a weak relationship between the [x, y] rank-ordered distance from the global minimum and the error (Aii). In contrast, Panels Bi, Ci display examples where there is a single error minimum in x and y, but with different properties at the edges; in Bi In both cases, the error monotonically increases with the distance from the single (global) minimum at [0, 0] (Bii, Cii), and we observe the impact of the gradient flat regions in Fig.Ci vs. Fig.Bi as the asymptote in Cii vs. Bii. These examples highlight that convexity is generally desirable because it produces a simple and well ordered landscape for optimization, as in B. However, C shows that a function can be nonconvex yet still retain a simple monotonic relationship between error and distance from the global minimum. Thus, for our application, a useful metric should distinguish the less well behaved landscape in A from the well ordered landscapes in B and C. In this sense, the property of interest is not strictly convexity per se, but whether error remains monotonically ordered with respect to distance from the global minimum. This monotonicity is quantified by Spearman’s Rank-Order Correlation (ρ), with higher values of ρ indicating that error is more consistently ordered by distance from the global minimum, and therefore indicating that a landscape is more amenable to optimization. Thus, in order to meta-learn an objective function with improved optimization properties, we use Spearman ρ between error (Err) and parameter set distance rank (DR), ρ(Err, DR) as the meta-learning target. In particular, we use a pattern search algorithm (Mayer *et al*., 2016) over ρ(Err, DR) to optimize the weights in the linear scalarization of the original multi-objective optimization (MOO) associated with the neuron model fitting problem.

## References

[R1] Allen Institute for Brain Science (2015) “Allen Cell Types Database.” http://celltypes.brainmap.org/.

[R2] Almog M. and Korngreen A. (2014) “A quantitative description of dendritic conductances and its application to dendritic excitation in layer 5 pyramidal neurons,” The Journal of neuroscience : the official journal of the Society for Neuroscience, 34(1), pp. 182–196. Available at: 10.1523/JNEUROSCI.2896-13.2014. 24381280 PMC6608163

[R3] Almog M. and Korngreen A. (2016) “Is realistic neuronal modeling realistic?,” Journal of Neurophysiology, 2(1), p. jn.00360.2016. Available at: 10.1152/jn.00360.2016. PMC5102320 27535372

[R4] Antic S.D. (2010) “The Decade of the Dendritic NMDA Spike,” Journal of neuroscience research, 88(14), pp. 2991–3001. Available at: 10.1002/jnr.22444. 20544831 PMC5643072

[R5] Awile O. (2022) “Modernizing the NEURON Simulator for Sustainability, Portability, and Performance,” Frontiers in Neuroinformatics, 16. Available at: https://www.frontiersin.org/articles/10.3389/fninf.2022.884046 (Accessed: September 12, 2022). 10.3389/fninf.2022.884046 PMC9272742 35832575

[R6] Bahl A. (2012) “Automated optimization of a reduced layer 5 pyramidal cell model based on experimental data.,” Journal of neuroscience methods, 210(1), pp. 22–34. Available at: 10.1016/j.jneumeth.2012.04.006. 22524993

[R7] Bano-Otalora B. (2021) “Daily electrical activity in the master circadian clock of a diurnal mammal,” eLife. Edited by Dulac C., Tessmar-Raible K., and Allen C.N., 10, p. e68179. Available at: 10.7554/eLife.68179. 34845984 PMC8631794

[R8] Beyer H.-G. and Schwefel H.-P. (2002) “Evolution strategies – A comprehensive introduction,” Natural Computing, 1(1), pp. 3–52. Available at: 10.1023/A:1015059928466.

[R9] Bhalla U.S. and Bower J.M. (1993) “Exploring parameter space in detailed single neuron models: simulations of the mitral and granule cells of the olfactory bulb,” Journal of Neurophysiology, 69(6), pp. 1948–1965. Available at: 10.1152/jn.1993.69.6.1948. 7688798

[R10] Burkitt A.N. (2006) “A Review of the Integrate-and-fire Neuron Model: I. Homogeneous Synaptic Input,” Biological Cybernetics, 95(1), pp. 1–19. Available at: 10.1007/s00422-006-0068-6. 16622699

[R11] Das I. and Dennis J.E. (1997) “A closer look at drawbacks of minimizing weighted sums of objectives for Pareto set generation in multicriteria optimization problems,” Structural optimization, 14(1), pp. 63–69. Available at: 10.1007/BF01197559.

[R12] Dauphin Y. (2014) “Identifying and attacking the saddle point problem in high-dimensional non-convex optimization.” arXiv. Available at: 10.48550/arXiv.1406.2572.

[R13] De Rainville F.-M. (2014) “DEAP: enabling nimbler evolutions,” ACM SIGEVOlution, 6(2), pp. 17–26. Available at: 10.1145/2597453.2597455.

[R14] Druckmann S. (2007) “A novel multiple objective optimization framework for constraining conductance-based neuron models by experimental data,” Frontiers in neuroscience, 1(1), p. 7. Available at: 10.3389/neuro.01.1.1.001.2007. 18982116 PMC2570085

[R15] Druckmann S. (2011) “Effective Stimuli for Constructing Reliable Neuron Models,” PLOS Computational Biology, 7(8), p. e1002133. Available at: 10.1371/journal.pcbi.1002133. 21876663 PMC3158041

[R16] Druckmann S. (2013) “A hierarchical structure of cortical interneuron electrical diversity revealed by automated statistical analysis.,” Cerebral cortex (New York, N.Y. : 1991), 23(12), pp. 2994–3006. Available at: 10.1093/cercor/bhs290. 22989582

[R17] Frye C.G. (2021) “Critical Point-Finding Methods Reveal Gradient-Flat Regions of Deep Network Losses,” Neural Computation, 33(6), pp. 1469–1497. Available at: 10.1162/NECO_A_01388. 34496389 PMC8919680

[R18] Garcia J.D. (2025) “Differential roles of NaV1.2 and NaV1.6 in neocortical pyramidal cell excitability,” eLife, 14. Available at: 10.7554/eLife.105696.2. PMC12263156 40662949

[R19] Gerber S. (2010) “Visual Exploration of High Dimensional Scalar Functions,” IEEE Transactions on Visualization and Computer Graphics, 16(6), pp. 1271–1280. Available at: 10.1109/TVCG.2010.213. 20975167 PMC3099238

[R20] Gidon A. (2020) “Dendritic action potentials and computation in human layer 2/3 cortical neurons,” Science, 367(6473), pp. 83–87. Available at: 10.1126/science.aax6239. 31896716

[R21] Goldman M.S. (2001) “Global structure, robustness, and modulation of neuronal models,” Journal of Neuroscience, 21(14), pp. 5229–5238. Available at: 10.1523/jneurosci.21-14-05229.2001. 11438598 PMC6762863

[R22] Golowasch J. (2002) “Failure of averaging in the construction of a conductance-based neuron model,” Journal of Neurophysiology, 87(2), pp. 1129–1131. Available at: 10.1152/jn.00412.2001. 11826077

[R23] Gouwens N.W. (2018) “Systematic generation of biophysically detailed models for diverse cortical neuron types,” Nature Communications, 9(1). Available at: 10.1038/s41467-017-02718-3. PMC5818534 29459718

[R24] Gyulassy A. (2008) “A Practical Approach to Morse-Smale Complex Computation: Scalability and Generality,” IEEE Transactions on Visualization and Computer Graphics, 14(6), pp. 1619–1626. Available at: 10.1109/TVCG.2008.110. 18989018

[R25] Happ H.C. and Carvill G.L. (2020) “A 2020 View on the Genetics of Developmental and Epileptic Encephalopathies,” Epilepsy Currents, 20(2), pp. 90–96. Available at: 10.1177/1535759720906118. 32166973 PMC7160871

[R26] Hausser M., Spruston N. and Stuart G.J. (2000) “Diversity and dynamics of dendritic signaling,” Science, 290(5492), pp. 739–744. Available at: 10.1126/science.290.5492.739. 11052929

[R27] Hay E. (2011) “Models of neocortical layer 5b pyramidal cells capturing a wide range of dendritic and perisomatic active properties.,” PLoS computational biology, 7(7), p. e1002107. Available at: 10.1371/journal.pcbi.1002107. 21829333 PMC3145650

[R28] Hedrich U.B.S., Lauxmann S. and Lerche H. (2019) “SCN2A channelopathies: Mechanisms and models,” Epilepsia, 60(S3), pp. S68–S76. Available at: 10.1111/epi.14731. 31904120

[R29] Hille B. (1984) Ionic channels of excitable membranes. Sunderland, Mass.: Sinauer Associates.

[R30] Hines M. (1984) “Efficient computation of branched nerve equations.,” International journal of bio-medical computing, 15(1), pp. 69–76. 6698635 10.1016/0020-7101(84)90008-4

[R31] Hines M. (1993) “The NEURON Simulation Program,” pp. 1–14.

[R32] Hodgkin A.L. and Huxley A.F. (1952) “A quantitative description of membrane current and its application to conduction and excitation in nerve,” Bulletin of mathematical biology, 117(1–2), pp. 25–71; discussion 5–23. Available at: 10.1113/jphysiol.1952.sp004764. 2185861

[R33] Hu C. (2009) “Protein kinase A activity controls the regulation of T-type CaV3.2 channels by Gbetagamma dimers.,” The Journal of biological chemistry, 284(12), pp. 7465–73. Available at: 10.1074/jbc.M808049200. 19131331 PMC2658042

[R34] Hu W. (2009) “Distinct contributions of Na(v)1.6 and Na(v)1.2 in action potential initiation and backpropagation,” Nature Neuroscience, 12(8), pp. 996–1002. Available at: 10.1038/nn.2359. 19633666

[R35] Izhikevich E.M. (2004) “Which Model to Use for Cortical Spiking Neurons?,” IEEE Transactions on Neural Networks, 15(5), pp. 1063–1070. Available at: 10.1109/TNN.2004.832719. 15484883

[R36] Keren N., Bar-Yehuda D. and Korngreen A. (2009) “Experimentally guided modelling of dendritic excitability in rat neocortical pyramidal neurones.,” J Physiol, 587(7), pp. 1413–37. Available at: 10.1113/jphysiol.2008.167130. 19171651 PMC2678217

[R37] Keren N., Peled N. and Korngreen A. (2005) “Constraining compartmental models using multiple voltage recordings and genetic algorithms.,” Journal of neurophysiology, 94(6), pp. 3730–42. Available at: 10.1152/jn.00408.2005. 16093338

[R38] Kessi M. (2020) “Intellectual Disability and Potassium Channelopathies: A Systematic Review,” Frontiers in Genetics, 11. Available at: 10.3389/fgene.2020.00614. PMC7324798 32655623

[R39] Kessi M. (2021) “Calcium channelopathies and intellectual disability: a systematic review,” Orphanet Journal of Rare Diseases, 16(1), p. 219. Available at: 10.1186/s13023-021-01850-0. 33985586 PMC8120735

[R40] Koch C. (1998) Biophysics of Computation: Information Processing in Single Neurons. Oxford University Press. Available at: 10.1093/oso/9780195104912.001.0001.

[R41] Kullmann D.M. (2002) “The neuronal channelopathies,” Brain: A Journal of Neurology, 125(Pt 6), pp. 1177–1195. Available at: 10.1093/brain/awf130. 12023309

[R42] Kullmann D.M. (2010) “Neurological Channelopathies.” Available at: 10.1146/annurev-neuro-060909-153122. 20331364

[R43] Ladd A. (2022) “Scaling and Benchmarking an Evolutionary Algorithm for Constructing Biophysical Neuronal Models,” Frontiers in Neuroinformatics, p. 58. 10.3389/fninf.2022.882552 PMC9248031 35784184

[R44] Mainen Z.F. (1996) “Electrotonic architecture of hippocampal CA1 pyramidal neurons based on three-dimensional reconstructions,” Journal of Neurophysiology, 76(3), pp. 1904–1923. Available at: 10.1152/jn.1996.76.3.1904. 8890303

[R45] Major G., Larkum M.E. and Schiller J. (2013) “Active Properties of Neocortical Pyramidal Neuron Dendrites,” Annual Review of Neuroscience, 36(1), pp. 1–24. Available at: 10.1146/annurev-neuro-062111-150343. 23841837

[R46] Marder E. and Goaillard J.-M. (2006) “Variability, compensation and homeostasis in neuron and network function,” Nature Reviews Neuroscience, 7(7), pp. 563–574. Available at: 10.1038/nrn1949. 16791145

[R47] Markram H. (2015) “Reconstruction and Simulation of Neocortical Microcircuitry,” Cell, 163(2), pp. 456–492. Available at: 10.1016/j.cell.2015.09.029. 26451489

[R48] Marler R.T. and Arora J.S. (2010) “The weighted sum method for multi-objective optimization: new insights,” Structural and Multidisciplinary Optimization, 41(6), pp. 853–862. Available at: 10.1007/s00158-009-0460-7.

[R49] Mayer A. (2016) “Diversity of immune strategies explained by adaptation to pathogen statistics,” Proceedings of the National Academy of Sciences, 113(31), pp. 8630–8635. Available at: 10.1073/pnas.1600663113. PMC4978245 27432970

[R50] Meliza C.D. (2014) “Estimating parameters and predicting membrane voltages with conductance-based neuron models,” Biological Cybernetics, 108(4), pp. 495–516. Available at: 10.1007/s00422-014-0615-5. 24962080

[R51] Mitchell M. (1998) An Introduction to Genetic Algorithms. MIT Press. Available at: https://books.google.com/books?hl=en&lr=&id=0eznlz0TF-IC&pgis=1 (Accessed: March 26, 2015).

[R52] Moody W.J. and Bosma M.M. (2005) “Ion Channel Development, Spontaneous Activity, and Activity-Dependent Development in Nerve and Muscle Cells,” Physiological Reviews, 85(3), pp. 883–941. Available at: 10.1152/physrev.00017.2004. 15987798

[R53] Nogaret A. (2022) “Approaches to Parameter Estimation from Model Neurons and Biological Neurons,” Algorithms, 15(5), p. 168. Available at: 10.3390/a15050168.

[R54] Oesterling P. (2013) “Visualizing nD point clouds as topological landscape profiles to guide local data analysis,” IEEE transactions on visualization and computer graphics, 19(3), pp. 514–526. Available at: 10.1109/TVCG.2012.120. 22566472

[R55] Otter N. (2017) “A roadmap for the computation of persistent homology,” EPJ Data Science, 6(1), p. 17. Available at: 10.1140/epjds/s13688-017-0109-5. 32025466 PMC6979512

[R56] Paninski L. and Cunningham J. (2018) “Neural data science: accelerating the experimentanalysis-theory cycle in large-scale neuroscience,” Current Opinion in Neurobiology, 50, pp. 232–241. Available at: 10.1016/j.conb.2018.04.007. 29738986

[R57] Prinz A.A., Bucher D. and Marder E. (2004) “Similar network activity from disparate circuit parameters.,” Nature neuroscience, 7(12), pp. 1345–52. Available at: 10.1038/nn1352. 15558066

[R58] Rall W. (1962) “Theory of Physiological Properties of Dendrites,” Annals of the New York Academy of Sciences, 96(4), pp. 1071–1092. Available at: 10.1111/j.1749-6632.1962.tb54120.x. 14490041

[R59] Rall W. (1992) “Matching dendritic neuron models to experimental data,” Physiological Reviews, 72(4 SUPPL.). Available at: 10.1152/physrev.1992.72.suppl_4.s159. 1438585

[R60] Ramaswamy S. (2015) “The neocortical microcircuit collaboration portal: a resource for rat somatosensory cortex,” Frontiers in Neural Circuits, 9(October), p. 44. Available at: 10.3389/fncir.2015.00044. 26500503 PMC4597797

[R61] Ranjan R. (2024) “eFEL.” Zenodo. Available at: 10.5281/zenodo.14222078.

[R62] Sakmann B. and Neher E. (1984) “PATCH CLAMP TECHNIQUES FOR STUDYING IONIC CHANNELS IN EXCITABLE MEMBRANES,” Ann. Rev. Physiol, 46, pp. 455–72. 6143532 10.1146/annurev.ph.46.030184.002323

[R63] Softky W.R. and Koch C. (1993) “The highly irregular firing of cortical cells is inconsistent with temporal integration of random EPSPs,” Journal of Neuroscience, 13(1), pp. 334–350. Available at: 10.1523/JNEUROSCI.13-01-00334.1993. 8423479 PMC6576320

[R64] Spratt P.W.E. (2019) “The Autism-Associated Gene Scn2a Contributes to Dendritic Excitability and Synaptic Function in the Prefrontal Cortex,” Neuron, pp. 1–13. Available at: 10.1016/j.neuron.2019.05.037. PMC7935470 31230762

[R65] Spratt P.W.E. (2021) “Paradoxical hyperexcitability from NaV1.2 sodium channel loss in neocortical pyramidal cells,” Cell Reports, 36(5). Available at: 10.1016/j.celrep.2021.109483. PMC8719649 34348157

[R66] Van Geit W. (2016) “BluePyOpt: Leveraging Open Source Software and Cloud Infrastructure to Optimise Model Parameters in Neuroscience,” Frontiers in Neuroinformatics, 10. Available at: 10.3389/fninf.2016.00017. PMC4896051 27375471

[R67] Vanier M.C. and Bower J.M. (1999) “A Comparative Survey of Automated Parameter-Search Methods for Compartmental Neural Models,” Journal of Computational Neuroscience, 7(2), pp. 149–171. Available at: 10.1023/A:1008972005316. 10515252

[R68] Wells S.A. (2024) “Inferring the Dynamics of Ionic Currents from Recursive Piecewise Data Assimilation of Approximate Neuron Models,” PRX Life, 2(2), p. 023007. Available at: 10.1103/PRXLife.2.023007.

[R69] Zitzler E. and Künzli S. (2004) “Indicator-Based Selection in Multiobjective Search,” in Yao X. (eds.) Parallel Problem Solving from Nature - PPSN VIII. Berlin, Heidelberg: Springer, pp. 832–842. Available at: 10.1007/978-3-540-30217-9_84.

